# A walk on the wild side: gut bacteria fed to mass-reared larvae of Queensland fruit fly [*Bactrocera tryoni* (Froggatt)] influence development

**DOI:** 10.1186/s12896-019-0579-6

**Published:** 2019-12-18

**Authors:** Lucas Alexander Shuttleworth, Mohammed Abul Monjur Khan, Terrence Osborne, Damian Collins, Mukesh Srivastava, Olivia Louise Reynolds

**Affiliations:** 1 Biosecurity and Food Safety, New South Wales Department of Primary Industries, Elizabeth Macarthur Agricultural Institute, Menangle, 2568 Australia; 2 Present address: NIAB EMR, Department of Pest and Pathogen Ecology, East Malling, Kent, ME19 6BJ UK; 3 Department of Entomology, Faculty of Agriculture, Bangladesh Agricultural University, Mymensingh, 2202 Bangladesh; 4 Graham Centre for Agricultural Innovation (an alliance between NSW Department of Primary Industries and Charles Sturt University), Wagga Wagga, New South Wales 2650 Australia; 5 State Key Laboratory of Ecological Pest Control for Fujian and Taiwan Crops, Institute of Applied Ecology, Fujian Agriculture and Forestry University, Fuzhou, 350002 China; 6 Present address: cesar Pty Ltd, Parkville, Victoria 3052 Australia

**Keywords:** *Asaia*, *Enterobacter*, *Lactobacillus*, *Leuconostoc*, Sterile insect technique, Area wide - integrated Pest management, Probiotic, Tephritidae, Diptera

## Abstract

**Background:**

The Queensland fruit fly, *Bactrocera tryoni* (Froggatt) (Diptera, Tephritidae) is the most significant insect pest of Australian horticulture. *Bactrocera tryoni* is controlled using a range of tools including the Sterile Insect Technique (SIT). Mass-rearing and irradiation of pupae in SIT can reduce the fitness and quality of the released sterile insects. Studies have also showed reduced microbial gut diversity in domesticated versus wild tephritids.

**Results:**

Transmission electron microscopy confirmed the presence of the bacterial isolates in the mid-gut of mass-reared larvae, and plate counts from individual larval guts showed increased numbers of bacteria in supplemented larvae. Several developmental and fitness parameters were tested including larval development time (egg-hatch to pupation), pupal weight, emergence, flight ability, sex-ratio, and time to adult eclosion (egg-hatch to adult eclosion). *Enterobacter* sp. and *Asaia* sp. shortened larval development time, while this was delayed by *Lactobacillus* sp., *Leuconostoc* sp. and a blend of all four bacteria. The mean time from egg hatch to adult eclosion was significantly reduced by *Leuconostoc* sp. and the blend for males and females, indicating that the individual bacterium and consortium affect flies differently depending on the life stage (larval or pupal). There was no impact of bacterial supplemented larvae on pupal weight, emergence, flight ability, or sex ratio.

**Conclusions:**

Our findings show that bacteria fed to the larval stage of *B. tryoni* can impart fitness advantages, but the selection of probiotic strains (individual or a consortium) is key, as each have varying effects on the host. Bacteria added to the larval diet particularly *Leuconostoc* sp. and the blend have the capacity to reduce costs and increase the number of flies produced in mass-rearing facilities by reducing time to adult eclosion by 1.3 and 0.8 mean days for males, and 1.2 and 0.8 mean days for females.

## Background

The Queensland fruit fly, *Bactrocera tryoni* Froggatt (Diptera, Tephritidae) is native to Australia, and is a pest and biosecurity threat to its $9 billion horticultural industry [1]. *Bactrocera tryoni* is controlled using a range of tools including the Sterile Insect Technique (SIT). SIT involves area-wide, inundative releases of irradiated, i.e. sterile insects to reduce reproduction in a wild population of the same species [2]. Domestication, mass-rearing and irradiation of pupae impact the quality of larvae and adult flies [3]. They also impact the tephritid gut microbiome, with flow on effects to physiology, behaviour and fitness [4].

Gut bacteria in particular have been recognised for their effects on the physiology of tephritids across all the developmental phases from egg to adult. These include increased larval weight of flies produced from surface sterilised eggs vs non-surface sterilised eggs [5], reduced larval development time [6], increased pupal weight [7, 8], larger males [8], preference of females to mate with bacteria fed males [9], improved male performance [10, 11], increased female fecundity [12], increased longevity [7, 8, 11, 13, 14], overcoming plant host defences [15], and insecticide resistance [16]. In addition to positive effects on tephritids that have been fed bacteria, there are also reported negative effects such as reduced pupation [17], decreased longevity of males [12], or neutral effects [6]. The majority of previous studies have focused on feeding bacteria to adults, resulting in limited information on the effects of bacterial supplementation at the larval stage. Furthermore, larval studies have predominantly focused on the family Enterobacteriaceae, likely due to the dominance of this family in the gut of several tephritids [4]. For example, a study feeding a blend of three enteric bacteria *Citrobacter freundii, Enterobacter* sp., and *Klebsiella pneumonia* to mass-reared Mediterranean fruit fly, *Ceratitis capitata* Wiedemann larvae (where male pupae were subsequently irradiated under SIT), showed increased male and female pupal weight, larger sized males, increased lab survival under stress, and enhanced male sexual performance [8]. Another enteric bacterial species, *Klebsiella oxytoca* increased mating competiveness of bacterial supplemented sterile adult male *C. capitata* for wild females against wild males, inhibited female receptivity more efficiently than sugar only fed males, and increased survival under stress [11]. A study that fed a single strain of *Enterobacter* sp. to *C. capitata* larvae reduced male larval development time, however did not impact pupal weight, flight ability, laboratory survival under stress, or mating competitiveness [6]. *Enterobacter* sp. was also a target probiotic fed to larvae of *Zeugodacus cucurbitae* Coquillett (melon fly) with significant increases in pupal weight, survival rate, and size of flies were significantly increased [7]. Although Enterobacteriaceae are dominant in several tephritids, other bacterial groups may play crucial roles [4]. Further research is therefore warranted to test the effects of feeding a greater diversity of bacterial strains to the larval stage.

The aims of the current study were to test the effects of bacteria sourced from wild larval *B. tryoni* and fed to mass-reared larvae, on larval development and several standard quality control parameters of the larval, pupal and adult stages used in SIT programs [18].

## Results

### Phylogenetic identification of wild *B. tryoni* bacterial candidates using 16S rRNA

**Fig. 1:**
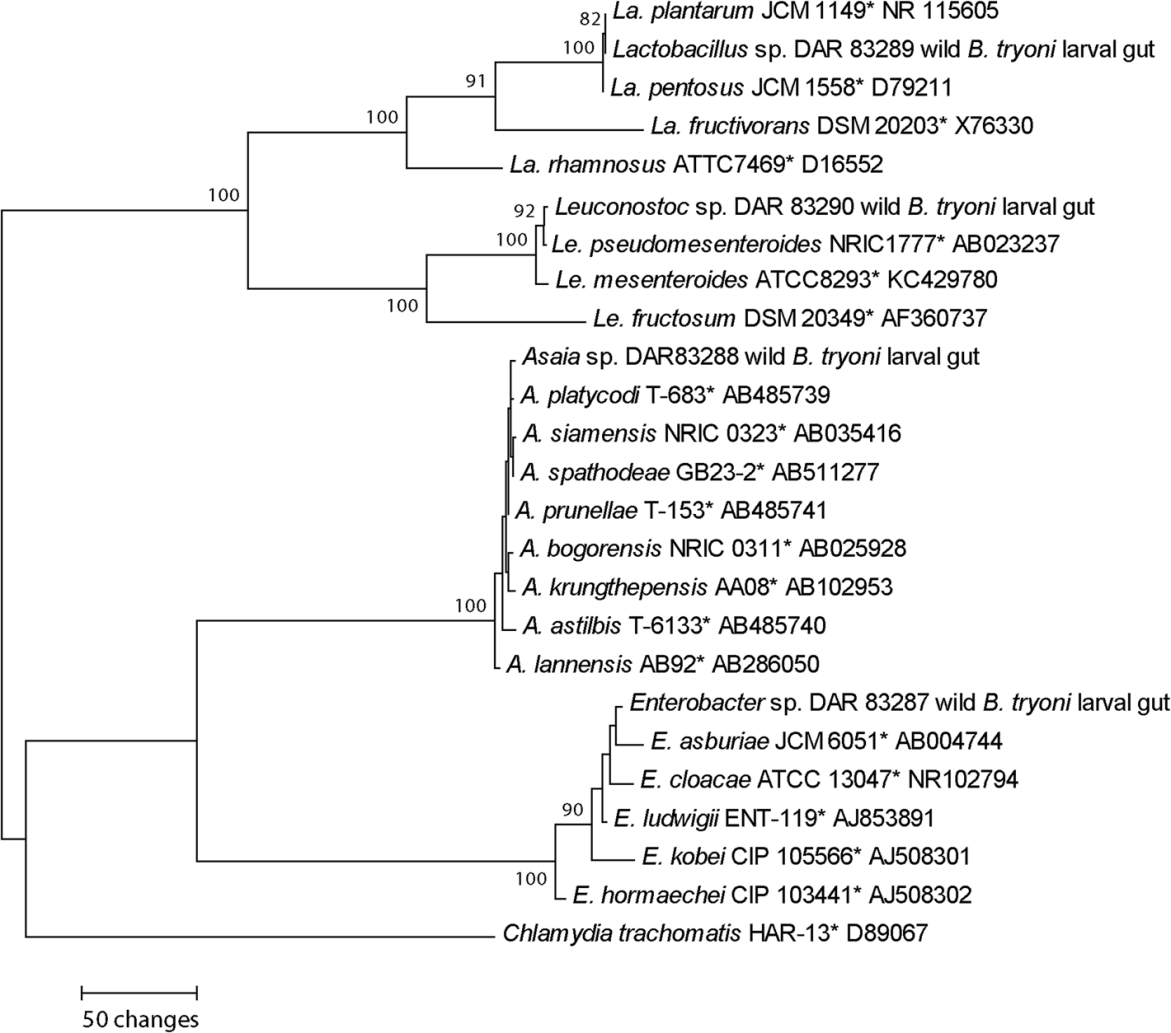
Maximum parsimony phylogeny using 16S rRNA sequences including wild bacterial candidates isolated from wild *B. tryoni* larval midguts, and fed to mass-reared larvae. *Chlamydia trachomatis* was selected as the outgroup. Maximum parsimony bootstrap supports ≥70% are placed on branch nodes. * indicates type culture. Culture and GenBank accessions are listed next to isolates

Phylogenetic analyses indicated that each bacterial strain isolated from the wild *B. tryoni* gut (*Asaia* sp. DAR 83288, *Enterobacter* sp. DAR 83287, *Lactobacillus* sp. DAR 83289 and *Leuconostoc* sp. DAR 83290) and utilised as a larval probiotic in this study, clustered with *Asaia* sp., *Enterobacter* sp., *Lactobacillus* sp. and *Leuconostoc* sp. clades respectively in the 16S rRNA maximum parsimony phylogeny (Fig. 1)*.*

### Quantification of bacterial cells in guts of mass-reared larvae fed wild bacteria

The mean number of colony forming units, isolated from third instar *B. tryoni* larvae, of each bacteria were higher than the control in both the supplemented individual and blend bacterial groups (all df = 12, *Asaia* sp.: F = 122.6, *p* < 0.001; *Enterobacter* sp.: F = 3282; *p* < 0.001; *Lactobacillus* sp.: F = 247.7; *p* < 0.001;), although this was not significant for *Leuconostoc* sp. (F = 3.17; df = 12; *p* = 0.078) (Table 1).

**Table 1:** The mean colony forming units isolated from individual mass-reared third instar larvae after feeding various bacteria supplements in the larval diet

Bacterial group/combination fed to mass-reared larvae	Mean colony forming units per larva	Standard error
Individual		
*Asaia* sp.	219,680	11,969
*Enterobacter* sp.	244,960	5653
*Lactobacillus* sp.	9980	2560
*Leuconostoc* sp.	18,720	14,071
Blend		
*Asaia* sp.	21,120	13,228
*Enterobacter* sp.	205,200	9156
*Lactobacillus* sp.	77	12
*Leuconostoc* sp.	9328	2194
Control (no added bacteria)		
*Asaia* sp.	45	5
*Enterobacter* sp.	1603	122
*Lactobacillus* sp.	4	1
*Leuconostoc* sp.	3236	1502

### Transmission electron microscopy of mass-reared larvae fed wild bacteria

**Fig. 2:**
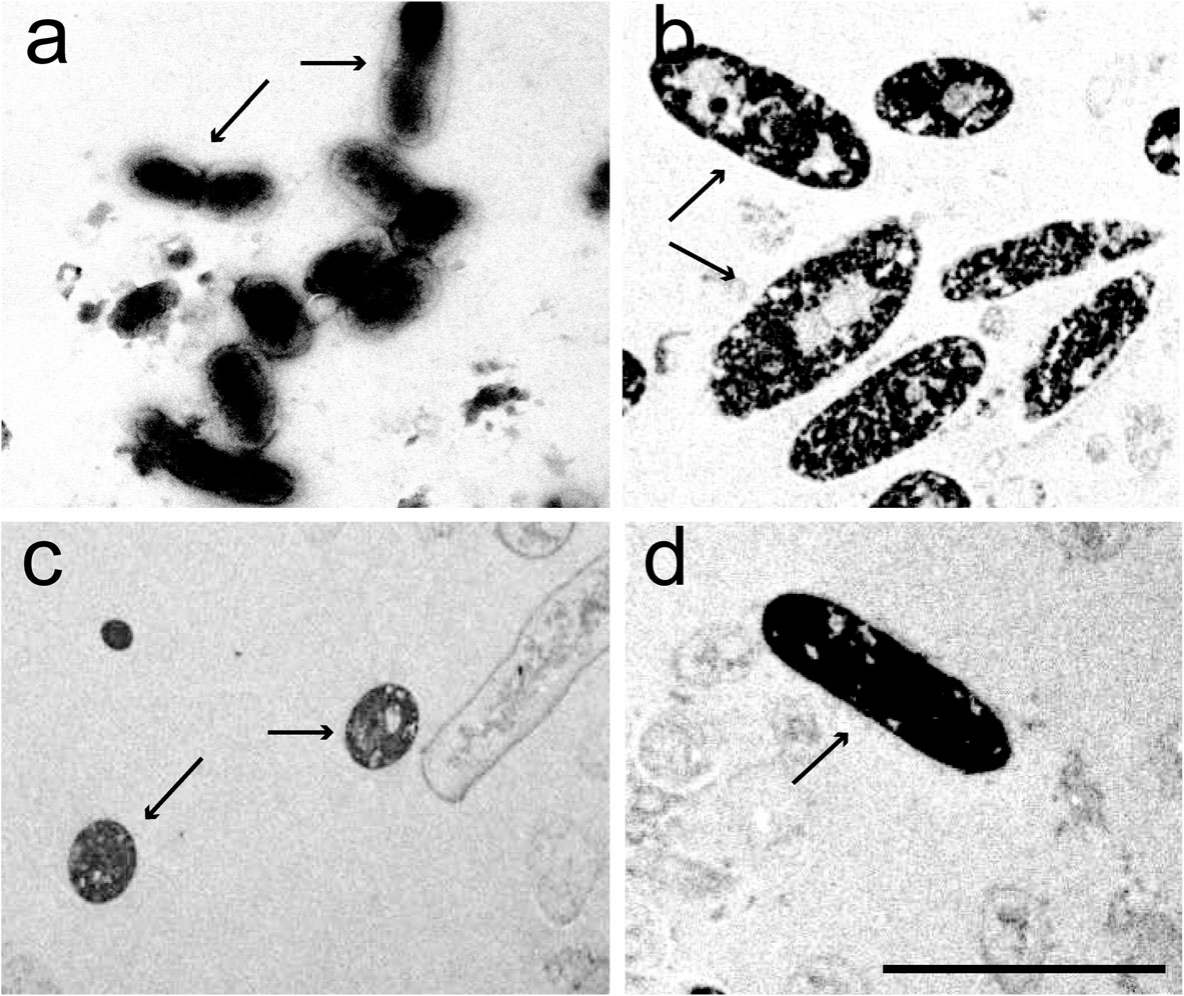
Transmission electron micrographs of bacterial cells after feeding to mass-reared *B. tryoni* larvae. **a**=, *Asaia* sp. cells, **b** = *Enterobacter* sp. cells, **c** = *Leuconostoc* sp. cells, **d** = *Lactobacillus* sp. cell. Arrows indicate cells from the specific strains. Scale = 5 μm

The bacteria provided to larval *B. tryoni* were visualised with transmission electron microscopy (Fig. 2), illustrating the presence of the bacteria within the third larval instar midguts after supplementation in the carrot diet.

### Larval development time

**Fig. 3:**
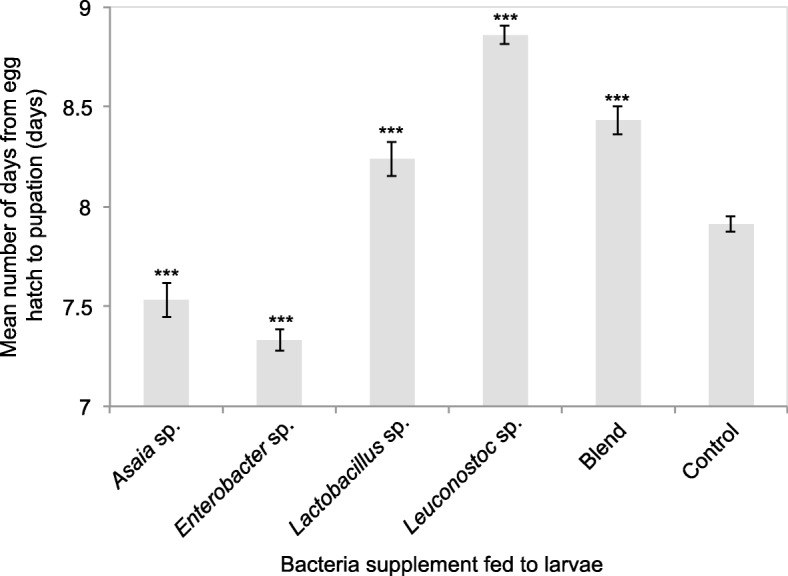
Time (days) from egg hatch to pupation (larval development) of *B. tryoni* larvae supplemented with various wild bacteria. Error bars indicate standard error of the mean. Asterixes above columns indicate if the bacterial group is significantly different to the control and the respective *p*-value (df = 55, * *p* < 0.05, ** *p* < 0.01, *** *p* < 0.001)

All bacterial supplemented larvae had significantly lower or higher larval development time (LDT) than the control (df = 55 F = 74.1 *p* < 0.001). *Enterobacter* sp. and *Asaia* sp. reduced LDT, while *Lactobacillus* sp., *Leuconostoc* sp. and the blend delayed this parameter (Fig. 3). *Asaia* sp. and *Enterobacter* sp. supplemented larvae had a mean LDT of 7.53 and 7.33 days, while *Lactobacillus* sp., *Leuconostoc* sp. and the blend supplemented had a mean LDT of 8.24, 8.86 and 8.43 days respectively.

### Pupal weight

**Fig. 4:**
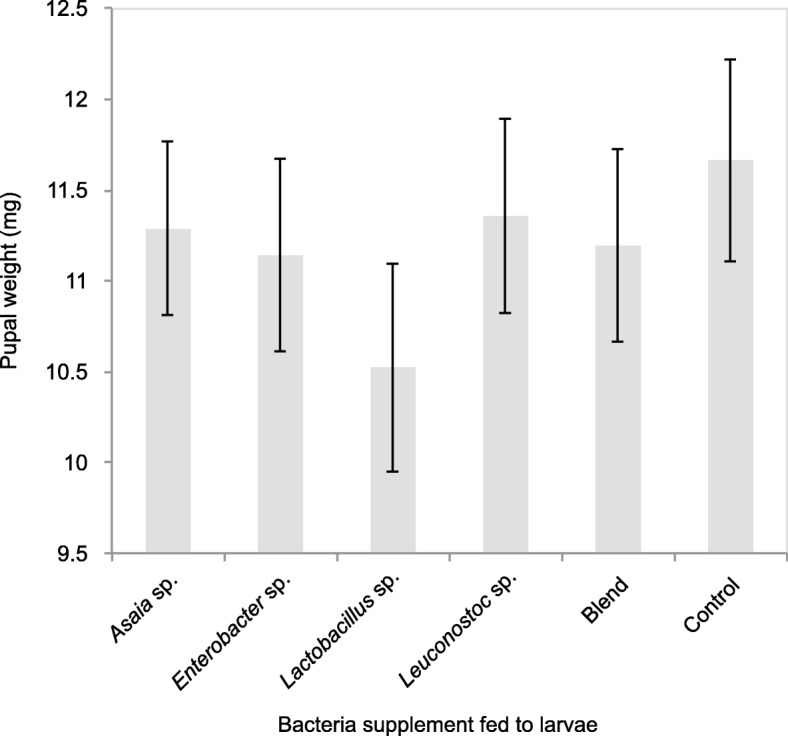
Mean pupal weight of mass-reared *B. tryoni* larvae supplemented with bacteria isolated from wild *B. tryoni* larvae. Error bars indicate standard error of the mean. The ANOVA showed there were no significant difference of pupal weight between the bacterial groups fed to larvae and the control (F = 1.97, df = 25, *p* = 0.118)

Pupal weight did not differ between the bacterial groups (Fig. 4; df = 25 F = 1.97 *p* = 0.118). However, the mean pupal weight of *B. tryoni* supplemented with the bacteria were all lighter than the control (mean individual pupa weight of 11.67 mg), with the lightest pupae those supplemented with *Lactobacillus* sp. (mean individual pupa weight of 10.52 mg).

### Adult eclosion

**Fig. 5:**
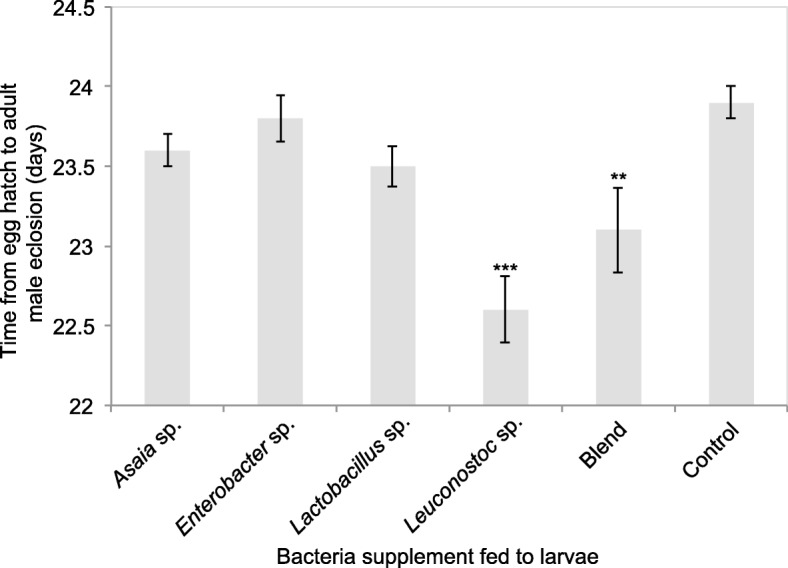
Time (days) from egg hatch to adult eclosion of mass-reared male *B. tryoni* in days supplemented with various wild bacteria as larvae. Asterixes above columns indicate if the bacterial group is significantly different to the control within each day and the respective *p*-value (* *p* < 0.05, ** *p* < 0.01, *** *p* < 0.001)

**Fig. 6:**
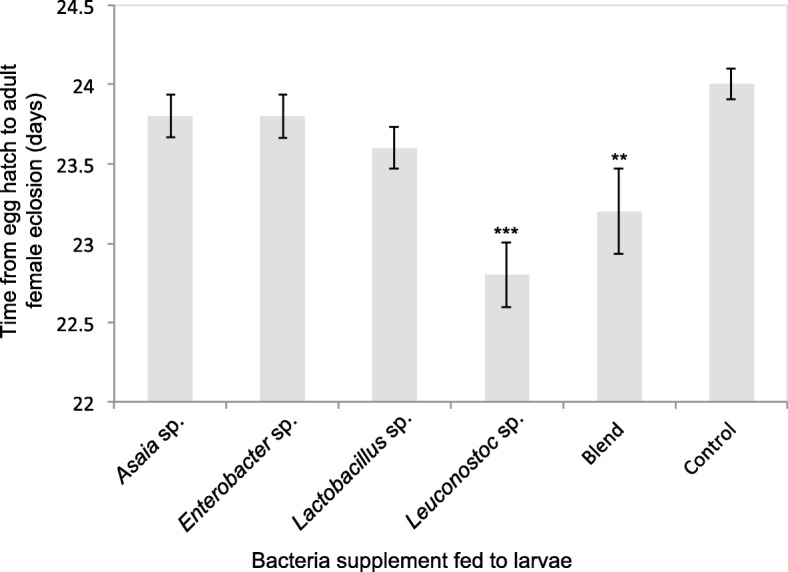
Time (days) from egg hatch to adult eclosion of mass-reared female *B. tryoni* in days supplemented with various wild bacteria as larvae. Asterixes above columns indicate if the bacterial group is significantly different to the control within each day and the respective *p*-value (* *p* < 0.05, ** *p* < 0.01, *** *p* < 0.001)

All bacteria supplemented *B. tryoni* larvae had a reduced mean period from egg hatch to adult eclosion in both males and females compared with the control (Figs. 5 and 6). The fastest eclosing *B. tryoni* males were those supplemented with *Leuconostoc* sp. or the blend, with the mean period from egg hatch to adult eclosion 22.6 days and 23.1 days respectively, compared to 23.9 days for the control males (df = 25, F = 6.1 *Leuconostoc* sp. *p* < 0.001, blend *p* < 0.01). The fastest eclosing *B. tryoni* females were also those supplemented with *Leuconostoc* sp. or the blend with 22.8 and 23.2 days respectively, compared to 24 days for the control females (F = 7.13 *Leuconostoc* sp. *p* < 0.001, blend *p* < 0.01).

### Emergence

The mean number of bacteria supplemented emerged adult *B. tryoni* did not differ to the control (df = 25 F1.09 *p* = 0.390). All bacterial groups had mean emergence ≥95% (Table 2).

**Table 2:** Emergence and flight ability of adult *Bactrocera tryoni* fed bacteria supplements in the larval diet

Bacteria group/combination	Mean emergence (%)	Standard error	Mean fliers (%)	Standard error
*Asaia* sp.	95a	2	91a	2
*Enterobacter* sp.	98a	1	93a	4
*Lactobacillus* sp.	98a	1	96a	2
*Leuconostoc* sp.	98a	1	96a	1
Blend	96a	1	93a	2
Control (no added bacteria)	97a	1	93a	3

Within each column, values followed by the same letter are not significantly different from one another (*p* > 0.05)

### Flight ability

The mean flight ability of adult *B. tryoni* supplemented with bacteria did not differ to the control (df = 25 F = 0.956 *p* = 0.463). Mean flight ability was ≥91% for all bacterial groups (Table 2).

### Sex ratio

There was no deviation from the expected sex-ratio of 1:1 for males and females produced by any of the bacteria supplemented larval diets (df = 25 F = 0.486 *p* = 0.782) (Table 2).

## Discussion

The present study demonstrated that wild bacteria supplemented larval *B. tryoni* influenced mass-reared larval development and adult eclosion, two parameters typically measured to assess the quality of flies used in SIT programs. The supplemented bacterial candidates were identified using near full-length 16S sequencing and phylogenetics and selected based on their known associations in the gut of wild larval *B. tryoni* [19]. Additionally, a *Lactobacillus* strain was selected based on the known associations of this genus in a diversity of animal species including insects, birds, rodents and humans [17, 20–22]. The *Enterobacter* sp. and *Asaia* sp. strains reduced larval development time, while this was delayed by *Lactobacillus* sp., *Leuconostoc* sp. and the blend. Conversely, the time from egg hatch to adult eclosion was significantly reduced by *Leuconostoc* sp., and the blend in both males and females, suggesting that pupal development was accelerated by these supplements. This showed that the varying bacteria affected *B. tryoni* development at different developmental stages, depending on the strain or consortium, and is probably linked with their function in the fly. Reductions in development times have been observed in the Mediterranean fruit fly (*Ceratitis capitata*) after supplementation with a strain of *Enterobacter* sp. in the larval diet, particularly of males [6]. Reductions in the tephritid development periods can increase efficiencies in the mass-rearing process and are key in large scale SIT operational programs [18]. High levels of productivity and faster development translate into cost efficiencies including production of higher numbers of flies per generation and reduction of the space required for mass-rearing.

In the current study, *B. tryoni* larvae supplemented with bacteria did not impact pupal weight, emergence, flight ability or sex ratio. Conversely, *Z. cucurbitae*, *Enterobacter* sp. fed to larvae was found to increase pupal weight [7], and a study on *C. capitata* larvae supplemented with a blend of *Citrobacter freundii*, *Enterobacter* sp. and *Klebsiella pneumoniae* also found an increase in pupal weight [8]. Another study on *C. capitata* with larvae fed a single strain of *Enterobacter* sp. found no difference in pupal weight [6]. In our study, pupal weight across all bacterial groups were all lighter than the control, however they were all above the 10 mg acceptable IAEA quality recommendation for *B. tryoni* [18]. In mass-reared *B. tryoni*, higher pupal weight has been found to be positively related to higher emergence and flight ability [23]. The three previously mentioned larval tephritid studies also analysed emergence and flight ability, and like the current study found no significant difference between the bacteria supplemented and control [6–8]. However there were effects on other parameters such as improved laboratory survival under water and food deprivation, increased adult fly size [7], and increased male mating competitiveness [8].

Previous tephritid bacterial supplementation assays have not included strains from the genera *Asaia, Leuconostoc,* and until recently *Lactobacillus.* Strains of *Asaia* are common insect symbionts [24] and have been shown to accelerate larval development of mosquitoes particularly by influencing the expression of host genes involved in cuticle formation [25, 26]. Strains in the genus *Leuconostoc* are not widely known from tephritids. In the few studies available they were identified from laboratory-reared and wild flies that were fed fruit at the larval stage [27–29]. Strains in the genus *Lactobacillus* affect several physiological and behavioural traits in tephritids and other Diptera. In a very recent study, *Lactobacillus plantarum* inoculated in to the larval diet of the tephritid *Dacus ciliatus* (cucurbit fly) was found to have negative effects on pupae production [17]. Conversely in *Drosophila melanogaster, L. plantarum* had positive effects including increased mating duration and induced higher short-term offspring production, and when fed to parent flies *L. plantarum* were reported to modulate body mass of female offspring [21]. This represented direct effects on adults as well as vertical effects. Further work is needed to fully elucidate the functional roles that probiotic bacteria have in tephritids.

The target bacteria were observed in the *B. tryoni* larval midguts by TEM after being fed the bacteria enriched carrot diet (Fig. 2), and the mean bacterial forming units isolated from larvae were higher when supplemented with both the individual bacteria and the consortium, compared to the control. A range of factors including pH, temperature, colonisation resistance of the gut (resistance to colonisation by non-indigenous species thus preventing infections from potential pathogens), redox conditions, digestive enzymes present and competition between bacteria in the diet, and/or within the larvae after ingestion may explain some of the observed differences in colony forming units between the different bacterial groups [30]. Indeed, the relatively lower counts of *Lactobacillus* sp. in the individual and blend supplemented larvae is probably due, at least in part, to colonisation resistance, as *Lactobacillus* sp. is a very minor component of the *B. tryoni* gut microbiome [19]. *Enterobacter* sp. appeared well adapted to the conditions and carrot diet used in the current experiment (pH 6, 26 °C). Similarly, the strain used proliferated in the larval gut, suggesting this environ is conducive to this bacteria. Most bacteria have an optimum pH 6–7 for growth, but several exceptions include lactic acid bacteria (e.g. *Lactobacillus* spp., *Leuconostoc* spp.) and acetic acid bacteria (e.g. *Asaia* spp). that can proliferate effectively under acidic environments [30] and are also likely to have different optimum temperatures for growth. *Lactobacillus plantarum* specifically has also been found to lower the pH of a tephritid larval diet from 5 to 4 after being added as a supplement [17]. Lactic acid bacteria and *Asaia* spp. are also common digestive tract associates of *B. tryoni* [19], and other insects including bees [31], beetles [32], mosquitoes [33, 34], and leaf hoppers [34]. Therefore in diets with low pH these bacteria would tend to proliferate quicker than those strains not adapted to low pH. Citric acid is a component of the standard carrot diet used at the former FFPF (Fruit Fly Production Facility, NSW Department of Primary Industries, Menangle) and other facilities, reducing pH to 4.5 [35]. The pH of the carrot diet used in the current study was 6 due to the omission of citric acid. This omission was made as some of the added bacteria were expected to lower the pH further [17] and therefore impact the development and fitness of larvae. The present study was conducted under a constant temperature of 26 °C, which is the optimum temperature determined for *B. tryoni* mass-rearing [18]. This temperature may not be ideal for all of the bacterial strains added to the diet. Closely related bacterial species do grow effectively over a wide range of temperatures [36–39], however the optimum growth temperatures of individual strains used as mass rearing supplements will need to be determined. In the laboratory we observed the four wild bacterial strains grew faster in culture at 30 °C, rather than 26 °C. The use of bacterial supplements in mass-rearing will need to consider optimal pH and temperature as factors when selecting appropriate probiotics to incorporate at the mass-rearing scale. Similarly, compatible consortiums also warrant further study.

## Conclusions

Bacteria fed to the larval stage of *B. tryoni* have scope to be administered as part of SIT programs to produce high quality insects. However, the selection of the bacterial strains is key as they each have specific effects, particularly on development time*.* Further studies need to address the effects of supplemented wild bacteria on other traits including survival and mating competitiveness of *B. tryoni* [40]. Ultimately, an understanding of the functional roles of individual bacterial strains in the insect gut and their use as larval probiotics, including as consortiums, is an area which requires increased focus, to better utilise and/or manipulate gut microbiota in pest management programs incorporating SIT.

## Methods

### Isolation of bacteria from wild *B. tryoni* larvae and culture deposit

Peaches infested with wild *B. tryoni* larvae were sourced from Redlands Research Station (RRS), Queensland. Infested apricots were sourced from Douglas Park, New South Wales. Individual third instar larvae had their midguts dissected and were lysed by hand with a sterile plastic mortar in 1 mL of sterile 1x Phosphate Buffered Saline (PBS). One hundred microliters of the resulting suspension was then spread on de Man, Rogosa and Sharpe agar (MRS; Becton, Dickinson and Company, MD, U.S.A.) and trytone soy agar (TSA; Becton, Dickinson and Company, MD, U.S.A.) plates in triplicate. Plates were incubated at 30 °C for 2–3 days. Single colonies were selected and subcultured on to MRS and TSA plates and incubated for 2–3 days at 30 °C. Pure cultures were deposited at the New South Wales Plant Pathology Herbarium, Orange Agricultural Institute, New South Wales, Australia (DAR) (Table 3).

**Table 3:** Culture accessions of wild bacteria used as probiotics fed to *B. tryoni* larvae

Species	Isolate	Location of collection	Host tree species
*Asaia* sp. (Acetobacteriaceae, Proteobacteria)	DAR 83288	Douglas Park, New South Wales	*Prunus armeniaca* (apricot)
*Enterobacter* sp. (Enterobacteriaceae, Proteobacteria)	DAR 83287	Redlands Research Station, Queensland	*Prunus persica* (peach)
*Lactobacillus* sp. (Lactobacillaceae, Firmicutes)	DAR 83289	Redlands Research Station, Queensland	*Prunus persica* (peach)
*Leuconostoc* sp. (Leuconostocaceae, Firmicutes)	DAR 83290	Redlands Research Station, Queensland	*Prunus persica* (peach)

DAR New South Wales Plant Pathology Herbarium, Orange Agricultural Institute, Orange, New South Wales, Australia

### Identification of bacterial isolates, RNA extraction, PCR, sequencing, phylogenetics and GenBank deposit

Purified subcultures were grown in the dark at 25 °C for 4 days on TSA (*Asaia* and *Enterobacter*) or MRS agar (*Lactobacillus* and *Leuconostoc*). RNA was extracted from a 5 mm^3^ sample of each isolate and placed in to 100 μL of Extraction Buffer (Sigma Aldrich, Australia), homogenised by hand with a sterile plastic probe for 10 s, then heated at 95 °C for 5 min. One hundred μL of Dilution Buffer (Sigma Aldrich, Australia) was then added. The 16S rRNA locus was selected for identifying the wild bacterial candidates to genus level. PCR amplification was performed in 10 μL final volumes. The primer pairs for amplification were FD1/rP2 or FD2/rP1 [41]. Each reaction contained 5 μL 2x MyTaq HS Mix (Bioline, Australia), 0.5 μL of each primer and 3 μL of sterile PCR grade water, and 1 μL of template. The thermocycling conditions were as follows; one denaturation step of 5 min at 95 °C, followed by thirty five cycles of 30 s at 95 °C, 30 s at 52 °C, and 45 s at 72 °C, followed by a final extension step of 5 min at 72 °C. Amplicons were visualised on a 1% agarose gel and sent to the Australian Genome Research Facility (Westmead, NSW) for Sanger sequencing using the same primer sets used in the amplification reactions. Sequences of reference taxa were sourced from GenBank. The alignment was completed with the MAFFT option of Geneious 7 (Biomatters, New Zealand) and edited manually. The phylogenetic tree was inferred using maximum parsimony in MEGA 7 [42]. Maximum parsimony bootstrap values ≥70% were placed at the nodes on the phylogenetic tree. Sequences generated in this study were deposited in GenBank under accessions MF370517-MF370520.

### Selection of bacterial candidates to feed to mass-reared larvae

Bacterial candidates from the genera *Asaia, Enterobacter,* and *Leuconostoc* were selected based on their known associations in the gut of wild *B. tryoni* [4]. Additionally, a *Lactobacillus* isolate was selected based on the known gut associations strains in this genus have in a diversity of animal species including insects (eg. tephritids), birds, rodents and humans [17, 20–22]. A blend of all four of the individual bacteria was also included to observe any effects of increasing the diversity of wild bacteria fed to the larvae.

### Preparation of carrot diet enriched with wild bacteria

*Bactrocera tryoni* larvae were reared on a standard diet comprising 338 g dehydrated carrot (bulking agent), 60 g of Torula yeast, 2.5 g sodium benzoate, and 600 ml of water [35]. Ingredients were heated to 80 °C for 5 min, covered and left to cool to room temperature. The pH of the diet was 6. Live *Asaia* sp. and *Enterobacter* sp. cultures were grown on TSA plates, while the *Lactobacillus* sp. and *Leuconostoc* sp. cultures were grown on MRS agar plates for 3 days at 30 °C. The choice to use live bacteria in the larval diet was based on the observation of advantages of feeding live vs dead bacteria to tephritid larvae [6]. Loopfuls (5 mm^3^) of the *Asaia* and *Enterobacter* cultures were transferred to 30 mL sterile TSB, while the *Lactobacillus* and *Leuconostoc* cultures were transferred to 30 mL sterile MRS broth. Cultures were placed in a shaking incubator for 24 h at 30 °C and 30 rpm. After incubation tubes were centrifuged for 5 mins at 4000 rpm. The broth supernatant was removed, and the pellet washed with 30 mL of PBS, and centrifuged for 5 mins at the same speed. The PBS supernatant was removed. Bacterial suspensions 1 × 10^8^ colony forming units per mL were made in PBS. Colony forming units were determined via ocular density at 600 nm wavelength with a Versa Max microplate reader (Molecular Devices, California, U.S.A). Ocular densities and their corresponding colony forming units were determined with serial dilutions and plate counts. For washed bacteria suspended in PBS at a concentration of 1–2 × 10^8^ colony forming units per mL the ocular densities were as follows: *Asaia* sp. = 0.1, *Enterobacter* sp. = 0.2, *Lactobacillus* sp. = 0.2, *Leuconostoc* sp. = 0.05.

### Source of mass-reared *B. tryoni* eggs and conditions for laboratory-based experiments

Mass-reared *B. tryoni* eggs were sourced from the FFPF, oviposited by 2 week-old adult flies raised on carrot diet (same ingredients as above except the addition of citric acid at 9 g per kilogram of diet). All laboratory based experiments were run at 26 °C ± 1 °C, 65% ± 5% relative humidity, and 10:14 light:dark cycle.

### Larval development time

Seventy-two rectangular plastic take away containers (500 ml volume) with lids were prepared. Lids of the plastic containers had a 5 × 8 cm hole cut in the top, and a piece of white ‘Swiss voile’ polyester fabric 3 cm wider and longer than the container was placed under the lid to prevent the escape of larvae from the container. Thirty grams of autoclaved vermiculite was mixed with 60 ml sterile water and added to the base of each container. The bacteria enriched carrot diet, and the carrot diet without bacteria (control) were weighed in to sterile 90 mm petri dishes. The agar plates with carrot diet were then placed over the vermiculite inside the take-away containers. One hundred and fifty mass-reared *B. tryoni* eggs per replicate were randomly selected, twelve replicates per bacterial group. Circular black filter paper 90 mm in diameter was cut into quarters and autoclaved. Each quarter was moistened with 0.5 mL sterile water. One hundred and fifty eggs were placed on top of the moistened filter paper. Each quarter of filter paper containing eggs was then inverted, so that the eggs were in direct contact with the carrot diet in the agar plates. The lids of the agar plates were placed on top of the diet to maintain humidity during the egg hatch period. After 2 days, the lids were removed. Larval development time was measured from the time of egg hatch to the day of pupation by counting the number of pupae on each day of pupation until all larvae had pupated. All larvae pupated over a six-day period.

### Quantification of bacterial cells within mass-reared larvae after feeding wild bacteria

Seven days after egg hatch (representing late third instar larvae) five individual larvae per bacterial group were surface disinfested in 70% ethanol for 1 min, then rinsed in PBS. Larvae were transferred to 200 μL PBS and homogenised by hand with a sterile plastic mortar. Serial dilutions were prepared (10X, 100X) of the original extract. Fifty microliters of the original extract and of each dilution were aliquoted on to a TSA plate for the *Asaia, Enterobacter,* blend and control groups, and an MRS plate for the *Lactobacillus, Leuconostoc,* blend and control groups. Plates were incubated at 30 °C for 2–3 days and the colonies that were morphologically identical (gram stain, cell morphology) to *Asaia, Enterobacter, Lactobacillus* and *Leuconostoc* were counted and subcultured. To confirm identification of colonies, representatives were selected and sequenced using the 16S sequencing protocol previously described.

### Tranmission electron microscopy for visualising bacteria in mass-reared larval guts after feeding wild bacteria

Larval midguts were cut into 2 mm sections and placed overnight in Karnovsky’s fixative. Sections were rinsed in 1× PBS for 5 min (repeated three times) and placed in 1% buffered Osmium tetroxide for 4 h on a shaker. Sections were rinsed in deionized water for 5 min (repeated three times), immersed in 2% uranyl acetate for 1 h, then dehydrated in an ethanol series starting from 30 to 100% and finally in acetone. Sections were immersed in 50% acetone/Spurr’s resin mixture and shaken for 1 h, then fixed in pure resin and placed at 70 °C for 30 min, embedded in resin filled moulds, and polymerized overnight at 70 °C. Resin blocks were trimmed and 70 nm sections placed on 300 mesh copper grids, stained with 2% uranyl acetate, followed by Reynold’s lead citrate, washed in deionized water, and blot dried. Sections were visualised under a Philips 208 transmission electron microscope.

### Pupal weight

Pupae were weighed individually 7 days after pupation, corresponding to the time that pupae in the FFPF are weighed for quality control purposes. Six replicates, each comprising 50 pupae per bacterial group were weighed, totalling three hundred pupae per bacterial group.

### Emergence, flight-ability, and sex ratio

One hundred and fifty pupae were selected with 6 replicates per bacterial group (total 900 pupae per bacterial group). Emergence and flight ability were calculated as mean percentages, and sex ratio was calculated as a ratio of the total pupae. Time to adult eclosion was determined by counting the number of adults (male and female) eclosing on each day for each bacterial group. Flight tubes were set up as in [18] with one flight tube containing one replicate placed in a single 30 cm^3^ mesh cage (Bugdorm, Taiwan). Fliers that escaped the flight tube were collected daily over 6 days, until no more flies left the flight tubes. The number of fliers, non-fliers, part-emerged, and deformed were recorded and sexed. Flight ability was calculated as the number of fliers of the total pupae per bacterial group.

### Adult eclosion

One hundred and fifty pupae were selected, 6 replicates per bacterial group (total 900 pupae per bacterial group) and placed in 30 cm^3^ Bugdorm cages (one cage per replicate). Pupae were counted on each day of eclosion and sorted by sex. All adults eclosed over a six-day period.

### Statistical analyses

R 3.3.3 [43] was used to analyse all data sets. A quasi-Poisson Generalised Linear Model was applied to larval development time and adult eclosion data sets. A quasi-Binomial model was applied to the emergence, flight ability, and sex ratio data sets. ANOVA was used to analyse the pupal weight data, and to test the quantification of bacterial cells within mass-reared larvae data (after log 10 transformation). All analyses were tested against the non-bacteria supplemented control.

## Data Availability

The datasets used and/or analysed during the current study are available from the corresponding author on reasonable request.
